# Microevolution within ST11 group *Clostridioides difficile* isolates through mobile genetic elements based on complete genome sequencing

**DOI:** 10.1186/s12864-019-6184-1

**Published:** 2019-10-30

**Authors:** Yuan Wu, Lin Yang, Wen-Ge Li, Wen Zhu Zhang, Zheng Jie Liu, Jin-Xing Lu

**Affiliations:** 1 State Key Laboratory of Infectious Disease Prevention and Control, National Institute for Communicable Disease Control and Prevention, Chinese Center for Disease Control and Prevention, Beijing, China; 2 Collaborative Innovation Center for Diagnosis and Treatment of Infectious Diseases, Hangzhou, China; 3 BGI-Shen zhen, main building, Beishan industry zone, Yan tian District, Shenzhen, China

**Keywords:** *Clostridioides difficile*, *tcdC* deletion, Mobile genetic elements, Complete whole genome sequencing, CRISPR spacers, Capillary electrophoresis-based PCR-ribotyping

## Abstract

**Background:**

Clade 5 *Clostridioides difficile* diverges significantly from the other clades and is therefore, attracting increasing attention due its great heterogeneity. In this study, we used third-generation sequencing techniques to sequence the complete whole genomes of three ST11 *C. difficile* isolates, RT078 and another two new ribotypes (RTs), obtained from three independent hospitalized elderly patients undergoing antibiotics treatment. Mobile genetic elements (MGEs), antibiotic-resistance, drug resistance genes, and virulent-related genes were analyzed and compared within these three isolates.

**Results:**

Isolates 10,010 and 12,038 carried a distinct deletion in *tcdA* compared with isolate 21,062. Furthermore, all three isolates had identical deletions and point-mutations in *tcdC*, which was once thought to be a unique characteristic of RT078. Isolate 21,062 (RT078) had a unique plasmid, different numbers of transposons and genetic organization, and harboring special CRISPR spacers. All three isolates retained high-level sensitivity to 11 drugs and isolate 21,062 (RT078) carried distinct drug-resistance genes and loss of numerous flagellum-related genes.

**Conclusions:**

We concluded that capillary electrophoresis based PCR-ribotyping is important for confirming RT078. Furthermore, RT078 isolates displayed specific MGEs, indicating an independent evolutionary process. In the further study, we could testify these findings with more RT078 isolates of divergent origins.

## Background

*Clostridioides difficile* has emerged as the leading cause of antimicrobial and health care-associated diarrhea in humans [1]. *C. difficile* is widespread in the environment and the gastrointestinal tracts of humans and animals [2, 3]. The population structure of *C. difficile* consists mainly of 6 clades, clade1–5 and clade C-I [4]. Hypervirulent PCR-ribotype 027 from clade 2 has caused outbreaks and transmission around the world [5]. RT078, contained in clade 5, is important in animal infections, and its incidence in cases of symptomatic human infection is increasing [6, 7]. There are at least 3 STs in clade 5, and 10 RTs (033, 045, 066, 078, 126, 127, 193, 237, 280, and 281) for ST11 [8, 9]. The high proportion of mobile genetic elements (MGEs) (about 11% in strain 630) contributes to the remarkable dynamic and mosaic genome of *C. difficile* [10]. Transposable and conjugative elements, plasmids, bacteriophages, and clustered regularly interspersed short palindromic repeat (CRISPR) elements are considered as the main MGEs and play important roles in horizontal gene transfer (HGT) of *C. difficile* [11–13].

In our previous study, we characterized three ST11 *C. difficile* isolates from elderly hospitalized patients with distinct RTs were reported [9]. Here, we continued our in-depth exploration of the genetic features and genomic differences among those three closely related isolates based on complete whole genome sequencing to provide a better understanding of the microevolution within the ST11 group of *C. difficile*, and help accurately identification of hypervirulent RT078.

## Results and discussion

### Genomic features of the three *C. difficile* isolates

**Fig. 1:**
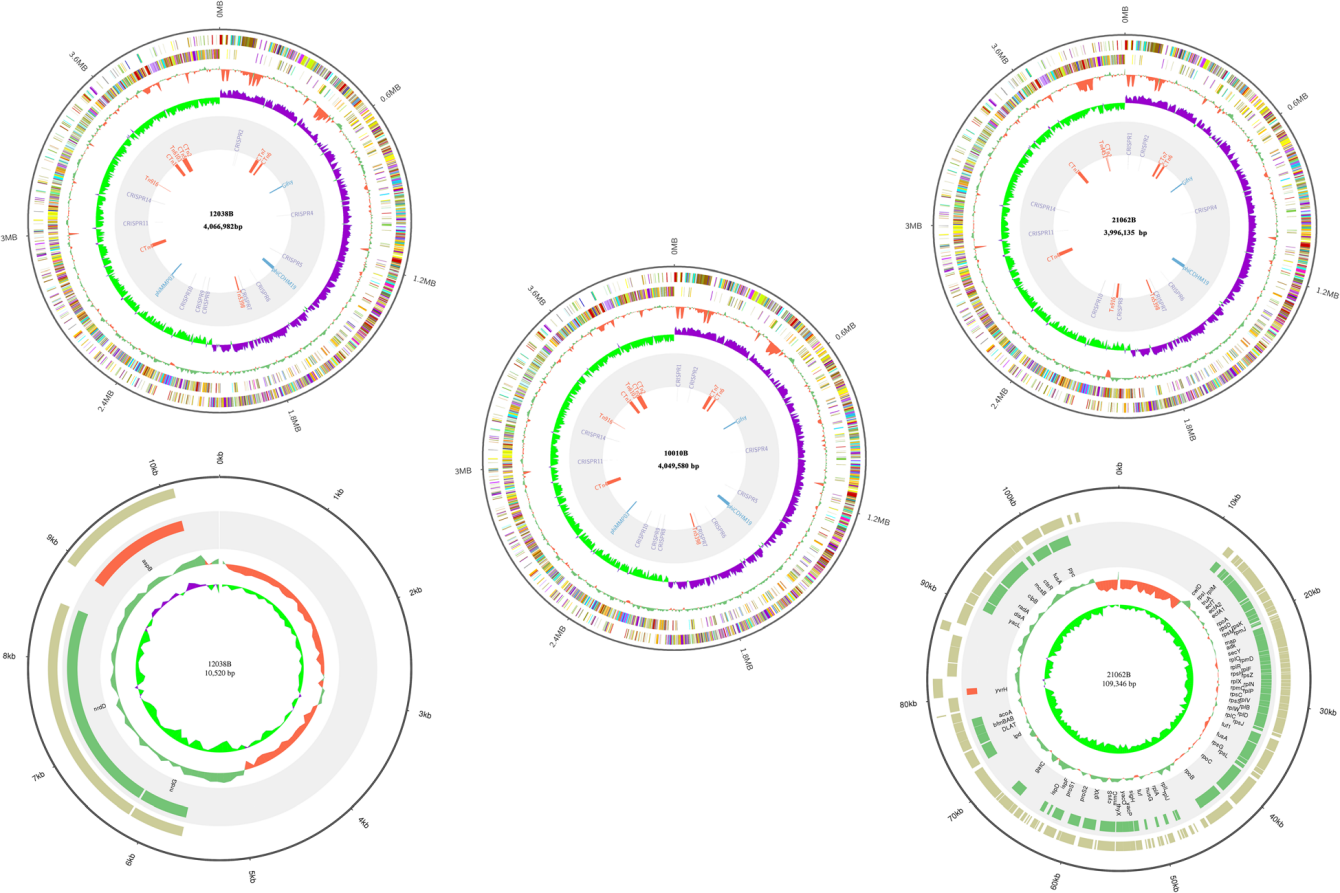
Schematic diagram of the complete whole chromosome and plasmid genomes of the three ST 11 *Clostridium difficile* isolates. For the chromosome genomes,, the circles (from the out layer inward) represent the genomes, the annotated COG genes on the positive strand, the annotated COG genes on the negative strand, GC content, GC skew, mobile genetic elements (red: the transposons; purple: the CRISPR; green: the prophages), and the name and genome size of the isolates, respectively. For the plasmid genomes,, the circles (from the outer layer inward) refer to GC skew, GC content, reverse strand genes, forward strand genes, all annotated genes and genome size

The three isolates 10,010 (new RT), 12,038 (new RT), and 21,062 (RT 078) used in this study have same MLST type (ST11) and toxin gene profile (*tcdA*^+^*tcdB*^+^*cdtA/B*^+^), however, in our previous study, we identified differences in PCR-ribotyping by capillary electrophoresis using the QIAXcel and ABT3730 systems [9]. The genome sizes of the three *C. difficile* isolates ranged from 3.99–4.07 Mb, of which isolate 21,063 had the fewest coding sequences (Table 1) (Additional file 1). The number and types of non-coding RNAs (ncRNA) and tandem repeats (TRs) are also summarized in Table 1. Schematic diagrams of the three complete chromosome genomes and two plasmid genomes are displayed in (Fig. 1). Isolates 12,038 and 21,062 carried one plasmid each (Fig. 1). Plasmid 12,038 had only 3 annotated genes, while plasmid 21,062 contained 69 genes, most of which encoded proteins involved in cell metabolism and transcriptional regulation. Furthermore, only one antibiotic-resistance gene, *rpoB* (associated with rifampicin resistance), was harbored on plasmid 21,062 (Fig. 1). For many bacteria, plasmids play an important role in drug resistance and are responsible for resistance transmission. However, in *C. difficile*, drug resistance genes are mainly carried on transposons not plasmid [12]. The first whole genome sequence of *C. difficile* was obtained for strain 630 and consists of a circular chromosome of 4.4 Mb and a plasmid, pCD630 of 7881 bp [10, 14]. Compared with strain 630, the three *C. difficile* isolates investigated in this study contained a smaller size of chromosomes with fewer coding sequences (Table 1 and Fig. 1). In addition, two plasmids identified in this study were larger than pCD630 (Fig. 1), which harbors 11 coding sequences (CDSs) with no obvious function. Importantly, CDSs carried by plasmid 21,062 and 12,038 were annotated as functional genes involved in many metabolic processes in *C. difficile* isolates, including the antibiotic resistance (Fig. 1).

**Table 1:** General feature of three ST 11 *C. difficile* isolates

Isolate	RT	Toxin gene	Origin	Age	Size Mp	CDS	tRNA	sRNA	TRF	Minisatellite DNA	Plasmids	Transposons	Prophage
10,010	new	A + B + CDT+	human	89	4.05	3624	89	52	481	367	0	CTn*1*, CTn*2*, CTn*4*, CTn*5*, CTn6*, CTn*7*, Tn*916*, Tn*6103*,Tn5398*	3
12,038	new	A + B + CDT+	human	89	4.07	3633	109	52	481	367	1	CTn*1*, CTn*2*, CTn*4*, CTn*5*, CTn6*, CTn*7*, Tn*6103*, Tn5398*	3
21,062	078	A + B + CDT+	human	92	3.99	3565	89	59	468	355	1	CTn*1*, CTn*4*, CTn6*, CTn*7*, Tn*5397*, Tn5398*, Tn*4453a*	2

### The genetic features of PaLoc and CdtLoc regions 3 ST11 *C. difficile* isolates

**Fig. 2:**
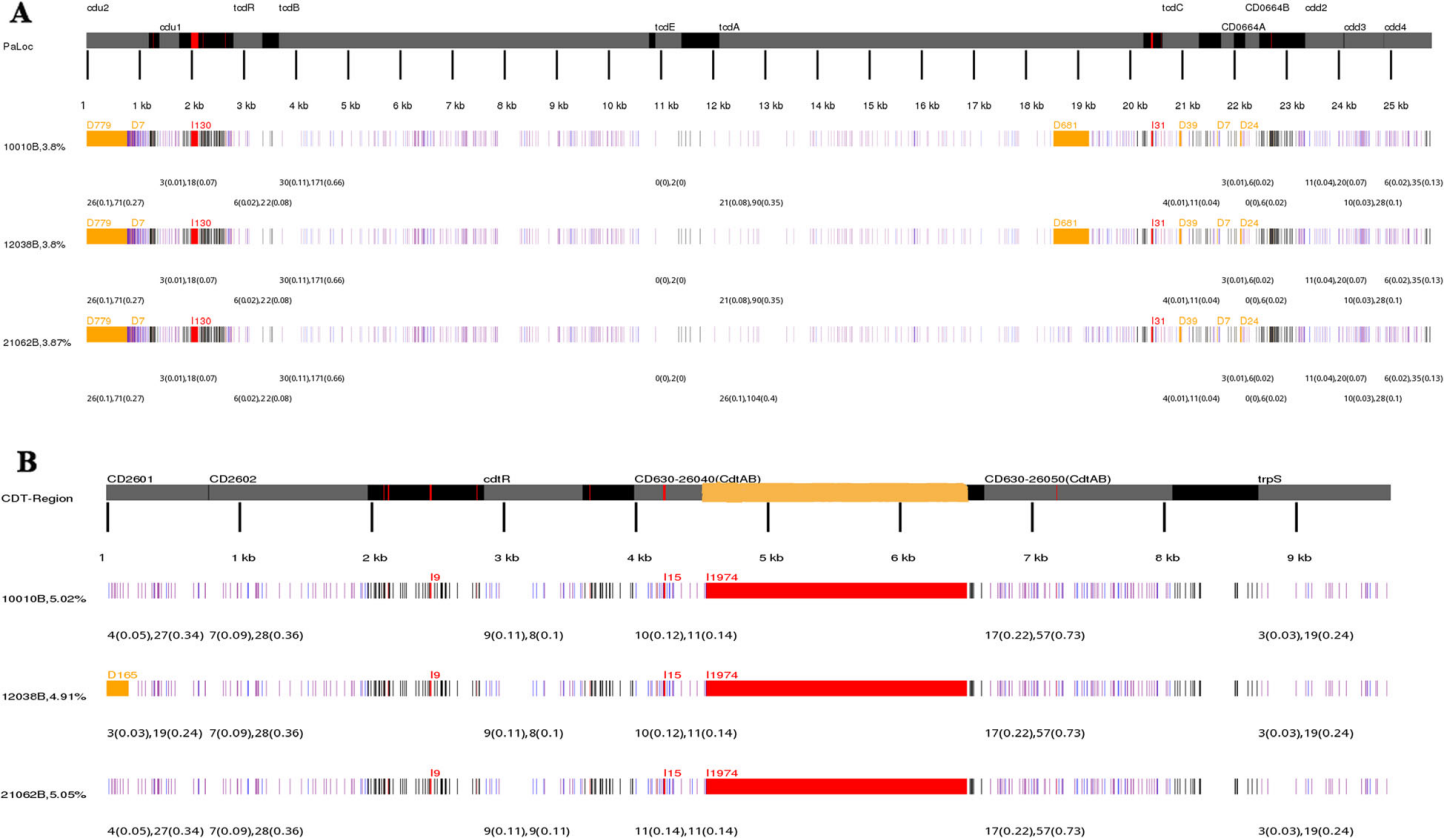
The sequence polymorphisms in the PaLoc and CdtLoc regions of the three ST11 *Clostridium difficile* isolates. CD630 was used as a reference. **a** Schematic representation of the PaLoc region and the polymorphisms within this area. **b** Schematic representation of the CdtLoc region and the polymorphisms within this area. The gray areas in the schematic representiations of the PaLoc and CdtLoc regions refers to coding genes, while the black areas refer to intergenic regions. Deletions are shown in orange, and insertions are shown in red. For example, D779 indicates a 779-bp deletion, and I130 indicates a 130-bp insertion. The numbers under each area indicate the number of synonymous mutations followed by the number of non-synonymous mutations. In addition, their proportions are shown in the brackets

All the three *C. difficile* isolates, which were *tcdA*^+^*tcdB*^+^*cdtA/B*^+^ positive, contained intact PaLoc and CdtLoc regions (Fig. 2). The PaLoc and CdtLoc regions among these isolates were almost identical (Fig. 2). Specifically, the location and length of deletions and insertions (indels) were the same, except the 661 bp deletion within *tcdA*, which was present only in isolate 10,010 and 12,038 (Fig. 2a). Compared with the other two isolates, isolate 21,062 contained a slightly greater number of single nucleotide polymorphism (SNPs), both synonymous and non-synonymous, within *tcdA* (Fig. 2a). However, the potential of this specific 661 bp deletion within *tcdA* as a unique marker of RT078 *C. difficile* remains to be confirmed in further studies of with more ST11 isolates. For CdtLoc region, the most significant characteristic was the intact *cdtA* and *cdtB* genes (with length of 6.2 kb) harbored by the three isolates (Fig. 2b), compared with truncated *cdtA* and *cdtB* gene (with length of 4.2 kb) in CD630 [10]. Moreover, the 165 bp deletion within the CD2601 coding region was found only in isolate 12,038 (Fig. 2b). The SNPs in *cdtR*, *cdtA*, *cdtB*, *trpS*, and intergenic regions in the three isolates were totally identical (Fig. 2b).

**Fig. 3:**
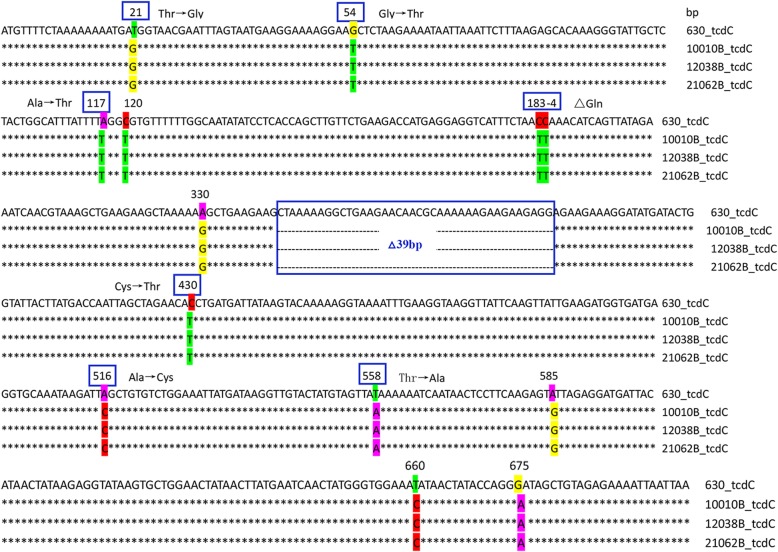
Partial sequence of the *tcdC* gene showing point-mutations and deletions. Base A, T, C, and G bases with mutations were shown in purple, green, red and yellow, respectively. The numbers in dark blue boxes above the base indicate the site of the mutations. The amino acid changes of caused by non-synonymous mutations are noted behind the mutation site. The △39-bp deletion is shown in a dark blue box

Importantly, a point mutation at position 184 and △39-bp deletion within *tcdC* has been reported as a specific feature of RT078 [15]. However, the △39-bp deletion was detected in all three ST 11 *C. difficile* isolates (Fig. 2a and Fig. 3). To explore the point mutations within *tcdC* in detail, the full length *tcdC* sequences from the three isolates were compared, which indicated that the point mutations were totally identical, including that at position 184 site leading to deletion of the amino acid Gln (Fig. 3). There were a total of 12 point mutations within *tcdC*, in which mutations at point positions 21, 54, 117,183–4, 430, 516, and 558 caused amino acid changes (Fig. 3). This result indicates that ST type together with toxin profile and deletions/mutation in *tcdC* cannot be used to confirm the hypervirulent RT078 *C. difficile* isolates. Identification of RT078 requires confirmation by PCR capillary electrophoresis, which is consistent with the findings of our previous study [9]. The *tcdC* gene encodes a negative regulator protein of toxins A and B in *C. difficile* [6, 16]. It is known that *tcdC* deletions lead to higher amounts of toxins A and B in RT027 [17]; however, the effect of the △39-bp deletion on the translation and expression of toxins in ST11 remains to be clarified.

### Analysis of the transposon and conjugative transposon in the three *C. difficile* isolates

A total of 11 types of transposons and conjugative transposons were identified in the three isolates (Table 2). Seven transposons reported in CD630 were all identified in the three isolates, although CTn*2* and CTn*5* were absent in isolate 21,062, and Tn*5397* was absent in isolate 10,010 and 12,038 (Table 1).

**Table 2:** Transposons and conjugative transposons analyzed in this study

Transposons	Referenced Isolates	Reference					Isolate 10,010		Isolate 12,038		Isolate 21,062		Common ORF^a^
		ORF	Size (kb)	Strat-end	GC%	Specific gene	ORF	enzymes	ORF	enzymes	ORF	enzymes	
CTn1	CD630	32	28.9	CD0355–0386	38.6	Xis, tyro-integrase	24	transposase	24	transposase	24	transposase	10
CTn2	CD630	36	42.2	CD0408–0436	35.1	seri-rebombinase	21	N	21	N	1	DNA helicase	13
Tn5397	CD630	19	20.7	CD0496–0511	38.3	tndX, tetM, group II intron	N	N	N	N	11	tetM	9
CTn4	CD630	28	30.5	CD1091–1118	46.6	Xis, int, transposase	28	Xis, int, transposase	28	Xis, int, transposase	28	Xis, int, transposase	13
CTn5	CD630	40	45.6	CD1845–1878	32.7		35		36		N	N	25
CTn6 (novel)	CD630	26	21.3	CD3326–3348	42.8	tyrointegrase	11	N	12	N	12	N	2
CTn7	CD630	30	29.2	CD3370–3392	40.9	seri-rebombinase	31	seri	31	seri	19	N	5
Tn6103	R20291		84.9	1740–1809	41.2	rebombinase							
Tn916		24	18			tetM, Xis, int	1	integrase	1	integrase	13	tetM, transposase	11
Tn5398	CD630	17	9.6	CD2001-2010b	35.4	ermB	7	N	7	N	7	N	5
Tn4453a/b	W1	7	6.3	Tnpx-tnpw		catD	1	helicase	1	helicase	10	N	3

^a^refers to ORFs found in the three isolates and reference CD630

**Fig. 4:**
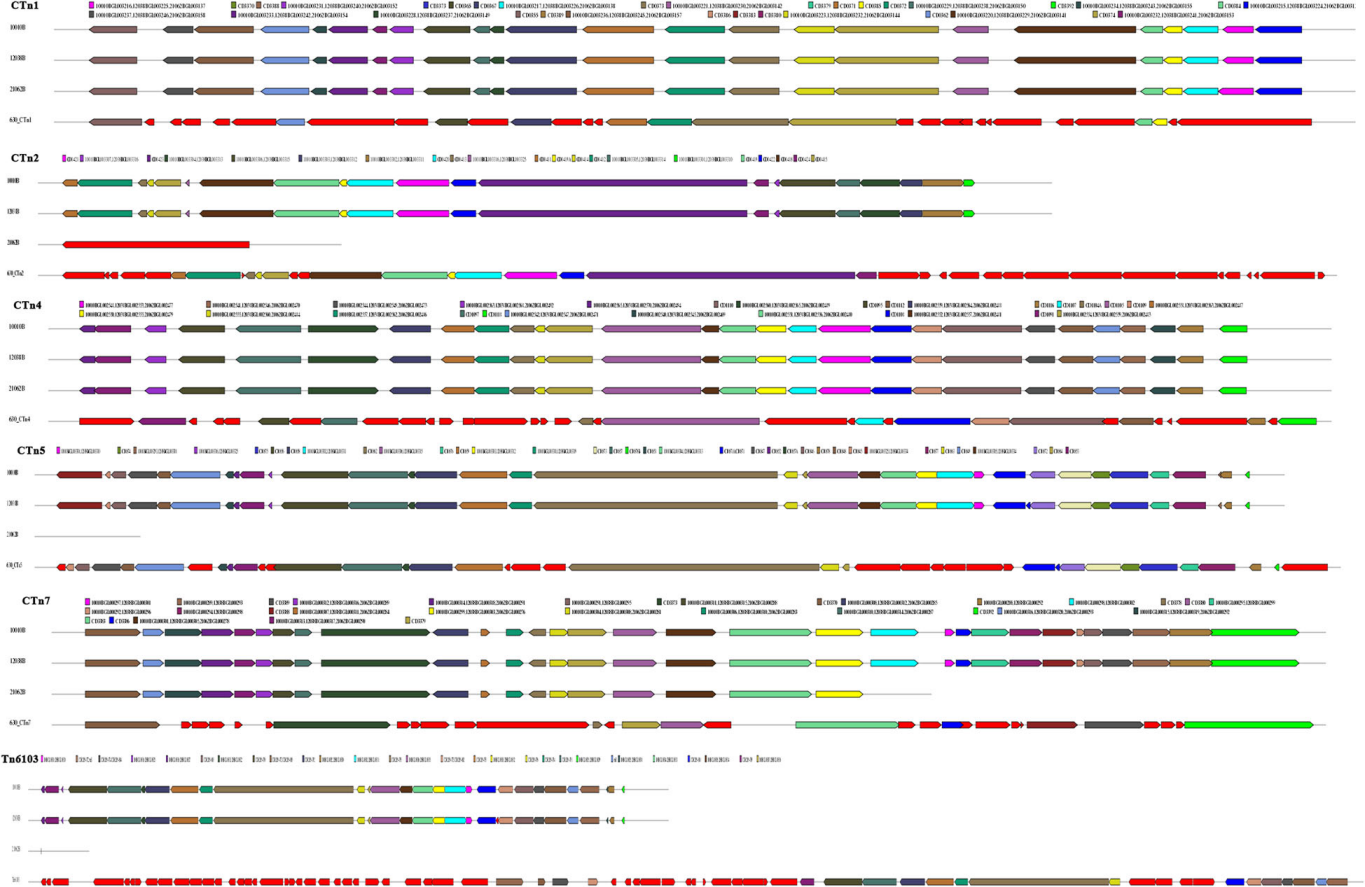
Schematic diagram of transposons and conjugative transposons (CTn*1*, CTn*2*, CTn*4*, CTn*5*, CTn*7*, and Tn*6103*) of the three ST11 *Clostridium difficile* isolates and the CD630 reference with relatively similar gene structure. Each open reading frame (ORF) is represented by a unique color. ORFs in red refer to a specific gene in each isolate. ORFs in the same color are recognized as the same coding genes

**Fig. 5:**
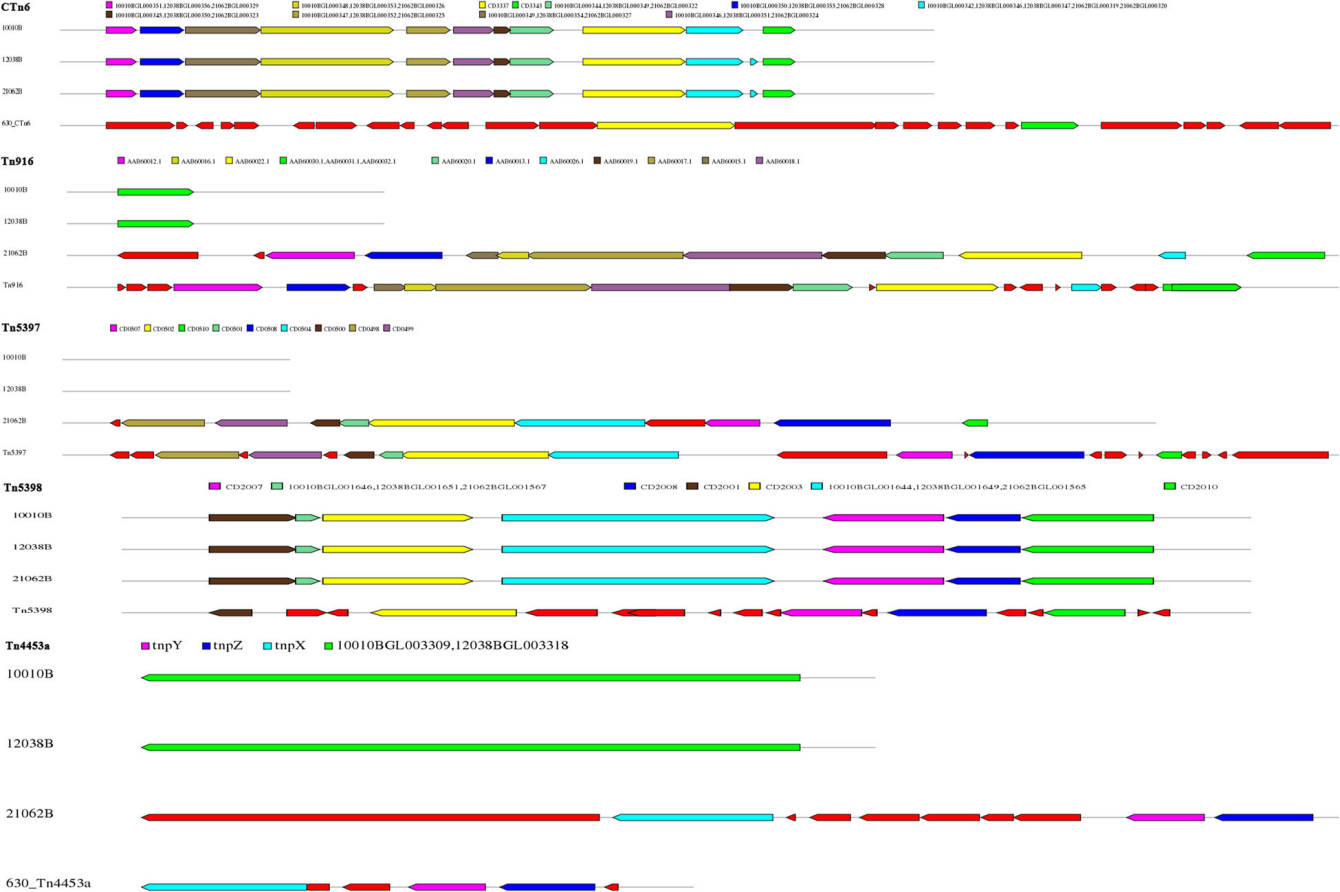
Schematic diagram of transposons and conjugative transposons (CTn*6*, Tn*916*, Tn*5397*, Tn*5398*, and Tn*4453a*) of the three ST11 *Clostridium difficile* isolates and CD630 reference with relatively similar gene structure. Each open reading frame (ORF) is represented by a unique color. ORFs in red refer to a specific gene in each isolate. ORFs in the same color are recognized as the same coding genes

CTn*1* has 32 ORFs in CD630, including a tyrosine integrase. CTn*1*-like elements in the three isolates were exactly the same as that in CTn*1* of CD630 but with fewer ORFs, the deletions of which were mainly existed in conjugative and accessary regions (Table 2 and Fig. 4). In addition, a transposase was found in these CTn*1*-like elements (Fig. 4). CTn*2*-like elements were detected only in isolates 10,010 and 12,038, but unlike the CT2 containing a serine recombinase, there was no transposase (Table 2 and Fig. 4). Only one open reading frame (ORF) encoding DNA helicase was retained in isolate 21,062 (Fig. 4). Tn*5397*, previously known as CTn*3*, was the first Tn*916*-like element to be extensively characterized in *C. difficile* [13]. This 21 kb element encodes tetracycline resistance via *tet*(M) and is highly related to Tn*916* across its length apart from the ends [4, 18], where two genes, *xisTn* and *intTn*, in Tn*916* are replaced by gene *tndX* in Tn*5397*. In this study, a Tn*5397*-like element found only in isolate 21,062 was devoid of *tndX* and a group II intron in *orf14*, while *tet*(M) was retained (Table 2 and Fig. 5). Due to the difference in gene composition between Tn*916* and Tn*5397*, Tn*916* has the ability to insert into multiple sites in the genome although it has a preferred consensus site, while Tn*5397* inserts into DNA predicted to encode a domain initially termed Fic (filamentation processes induced by cAMP) [19]. Bi-directional horizontal gene transfer of Tn*5397* between *C. difficile* strains and *E. faecalis* JH2–2, has been recently demonstrated [20]. However, the ability of the Tn*5397*-like element identified in this study to transfer between *C. difficile*, and other isolates, requires further investigation. CTn*4*-like elements with identical gene structure and order were detected in all three isolates (Fig. 4), and contained *xisTn* and *intTn* as detected in CTn*4* of CD630 (Table 2). CTn*5* is a Tn*1549*-like element and undergoes excision from the host genome at a transfer frequency of 2.8 × 10^− 5^ [18, 21]. In this study, CTn*5*-like elements with almost identical gene composition were only found in isolates 10,020 and 12,038 (Fig. 4). CTn*6* harbors a tyrosine integrase gene but without the excision ability. The novel elements identified in the three ST11 isolates in this study carried only two homologous genes (CD3337, encoding a membrane protein, and CD3343, encoding an AraC family transcriptional regulator) with CTn*6* (Fig. 5). Although there were no transposase genes, the novel element contained several genes encoding an ABC transporter in. The significance of CTn*7* is the presence of a large serine recombinase. CTn*7*-like elements with completely identical gene composition and order were identified in isolates 10,010 and 12,038 (Fig. 4). Interestingly, the CTn*7*-like element in isolate 21,062 was devoid of nearly one-third of the ORFs compared with the other two isolates, including the transposase homologous with CTn*7*, and seven flagella encoding genes (Fig. 4), although the impact of this on the flagella production and movement of isolate 21,062 (RT078) compared with isolates 10,010 and 12,038 remains to be determined. Tn*916* is one of the two largest families of conjugative transposons in *C. difficile*, carrying 24 potential ORFs, including *tet*(M), *xisTn* (an excisionase) and *intTn* (a tyrosine integrase), responsible for tetracycline resistance, excision, circularization and integration of the element [22]. In this study, a Tn*916*-like element retaining the *tet*(M) and transposase was identified only in isolate 21,062, while in isolate 10,010 and 12,038, there was only one ORF encoding an integrase (Fig. 5). Tn*5398* is a particular element in *C. difficile*, having no transposase, no circular form, but having an *oriT* site and two copies of the *ermB* genes [13]. Tn*5398* had been reported to transfer between *C. difficile* strains and from *C. difficile* to *Staphylococcus aureus* and *Bacillus subtilis* [23]. All three isolates in this study carried a Tn*5398*-like element was found to be absent with *ermB* genes and other potential genes (Fig. 5). The very large Tn*6103* (84.9 kb) was first recognized in strain R20291 (RT027) [12]. Although this element shows highly similarity with CTn*5*, there are three insertions of putative mobilizable transposons, designated Tn*6104*, Tn*6105* (both 15 kb and inserted into CD1743), and Tn*6105* (10 kb inserted into CD1776b) [13]. A Tn*6103*-like element was found in isolate 10,010 and 12,038, losing the whole Tn*6104* and almost the entirely Tn*6105* (Fig. 4). Tn*4453a/b* is the smallest element with only 7 ORFs in strain W1, of which the representative feature is carrying the gene *catD* [24]. A Tn*4453a/b*-like element was identified only in isolate 21,062 but without the gene *catD* gene, which was replaced by *aac* (21062BGL003409) (Fig. 5). Only one ORF encoding a helicase was found in isolate 10,010 and 12,018 (Fig. 5). It is known that *aac* encodes a bi-functional AME, accounting for more than 90% of high level gentamicin resistance (HLGR) in *E. faecalis* and *E. faecium* [25]. In our previous study of clade 4 *C. difficile* isolates, the same replacement in Tn*4453a/b* was also identified in some ST81/RT017 isolates (manuscript under review). However, the situation that promotes this replacement and whether this newly reported Tn*4453a/b* is transferred between intestinal bacteria as a complete element remain to be determined.

Transposons play an important role in the transfer of drug-resistance gene within *C. difficile* isolates, and between *C. difficile* and other bacteria, and in the genome re-construction, resulting in distinct phenotype in *C. difficile*. In this study, the RT078/ST11 isolate contained totally different transposon elements compared with the ST11 non-RT078 isolates. This indicates that these closely related isolates underwent distinct evolutionary processes, with RT078 derived from specific division pathway.

### CRISPRs reveal potential evolution pathways of the 3 ST11 *C. difficile* isolates

**Fig. 6:**
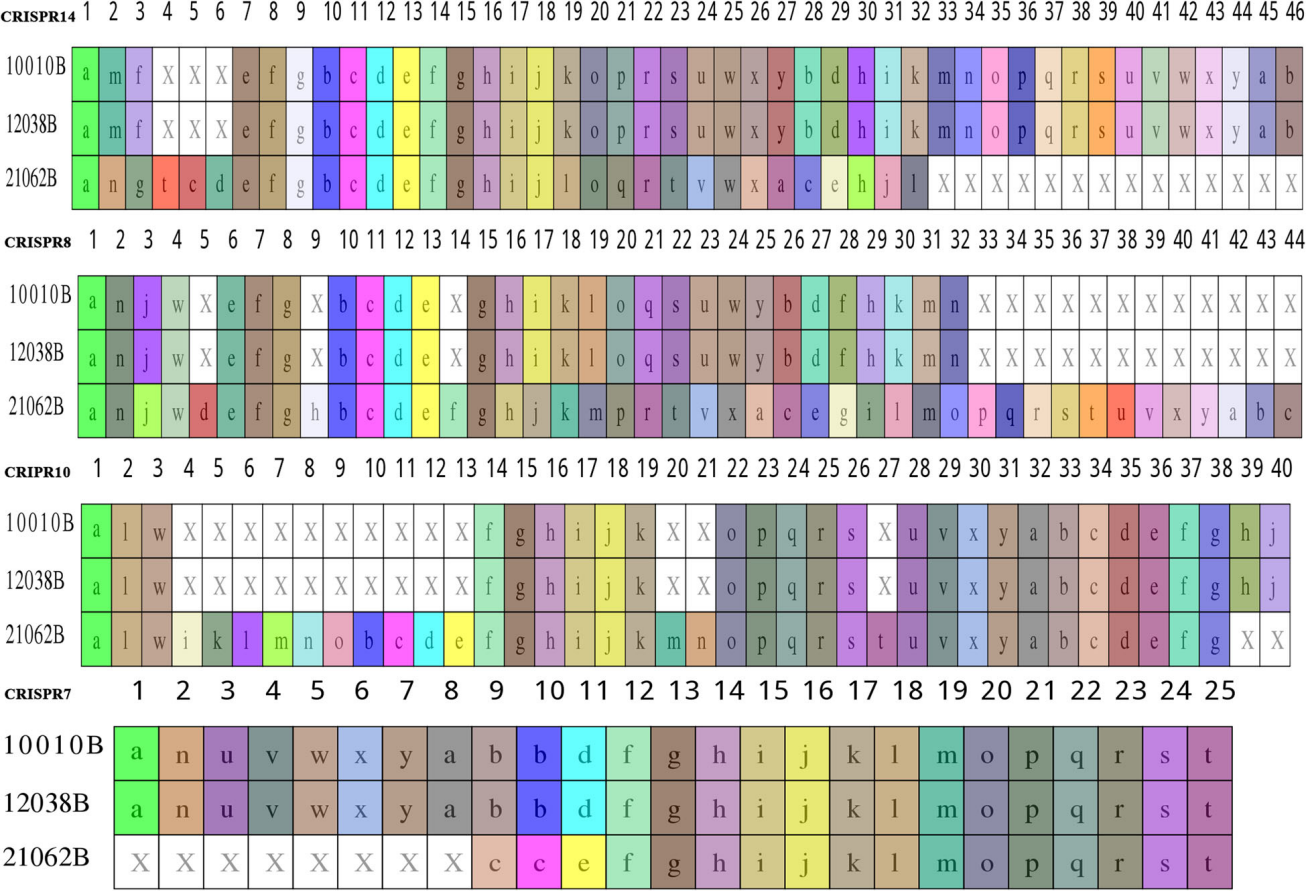
Schematic diagram of the composition of spacers (> 20) of the three ST11 *Clostridium difficile* isolates. A total of 14 types of CRISPR arrays were detected. Each box represents a spacer. Letters in the box refers to the length of the spacer, and X in the white box indicates deletion of this spacer. Boxes of the same color refer to totally identical spacers. The ancient spacer is located on the right

**Fig. 7:**
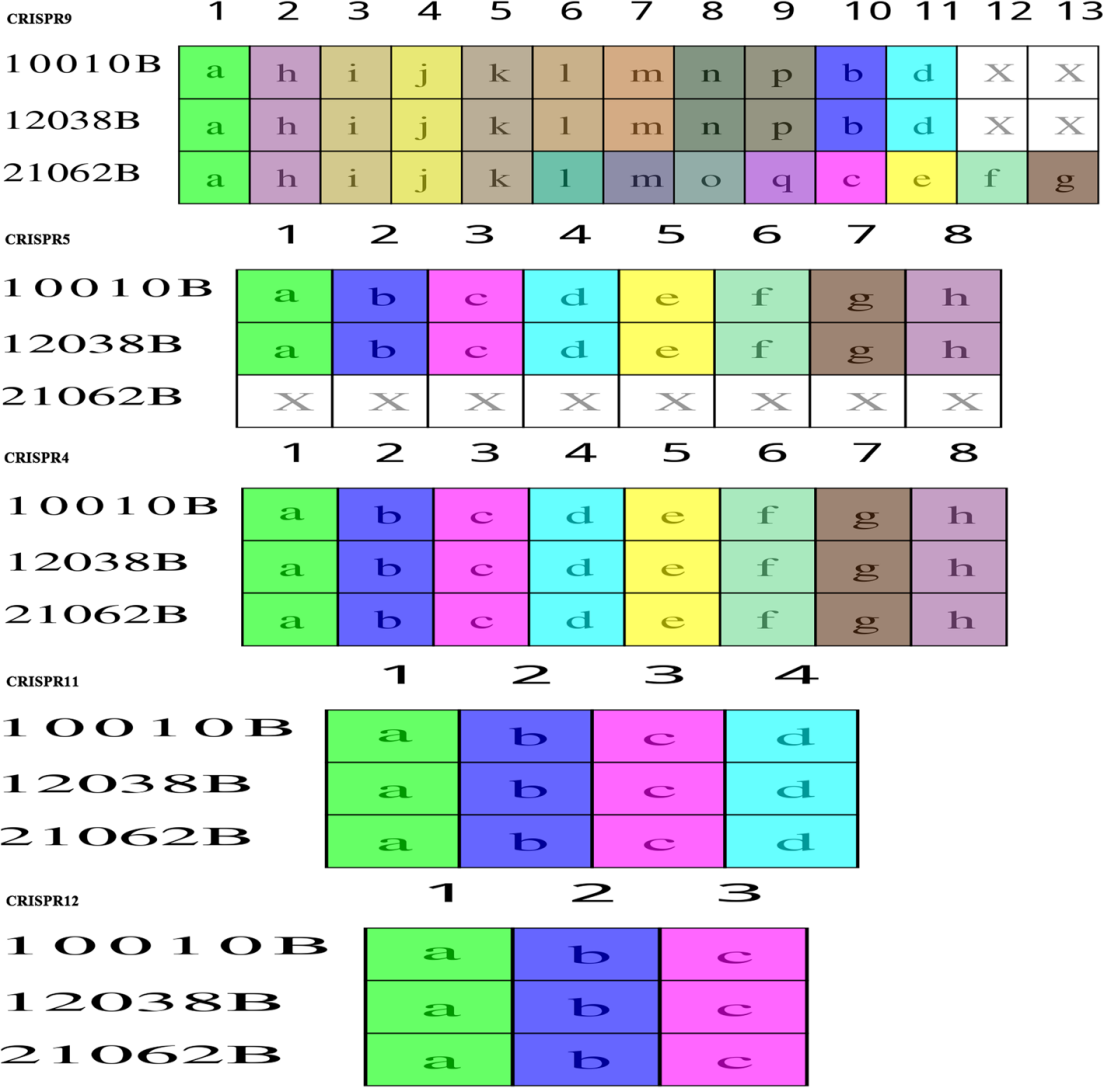
Schematic diagram of the composition of spacers (< 20) of the three *Clostridium difficile* isolates. Each box represents a spacer. Letters in the box refers to the length of the spacer, and X in the white box indicates deletion of this spacer. Boxes of the same color refer to totally identical spacers. The ancient spacer is located on the right

In searches of these 3 isolates, 13, 14, and 12 CRISPR arrays were identified in isolates 10,010, 12,038, and 21,062, respectively. Among the 14 arrays in 12,038, one was located in a plasmid. Based on subsequent comparison and classification of those arrays, a total of 14 types of CRISPR arrays were determined; these were designated CRISPR1–14 (Table 3). CRISPRs 1, 2, 3, 6, and 13 contained only one spacer that is identical within the isolates carrying them (Table 3). However, the distribution of CRISPRs 1, 2, 3, 6, and 13 among the three isolates was distinct, for example, CRISPR1 was absent from isolate 12,038, which was the only strain harboring CRISPR3 (Table 3). The remaining CRISPRs are shown as two groups with various numbers of spacers in Figs. 6 and 7. Identical CRISPRs with more than one spacer were detected in isolates 10,010 and 12,038 (Table 3, Figs. 6 and 7). Importantly, CRISPRs identified in isolate 21,062 (RT078) were distinct from those in the other two isolates (Figs. 6 and 7). Specifically, CRISPRs 3 and 5 were absent, and furthermore, in CRISPRs 7–10 and 14, there was great variation in the number and length of spacers, with numerous deletions and insertions of specific spacers (Figs. 6 and 7). In addition, CRISPRs 2, 4, 6, 11, 12, and 13 contained identical spacers in the three ST11 isolates, but with different RTs (Table 3, Fig. 7).

**Table 3:** The 14 CRISPRs identified in three isolates

Isolate	CRISPR name													
	1	2	3	4	5	6	7	8	9	10	11	12	13	14
10,010 (162)	√(1)	√(1)	×(0)	√(8)	√(8)	√(1)	√(25)	√(29)	√(11)	√(27)	√(4)	√(3)	√(1)	√(43)
12,038(162)	×(0)	√(1)	√(1)	√(8)	√(8)	√(1)	√(25)	√(29)	√(11)	√(27)	√(4)	√(3)	√(1)	√(43)
21,062(163)	√(1)	√(1)	×(0)	√(8)	×(0)	√(1)	△(17)	△(44)	△(13)	△(38)	√(4)	√(3)	√(1)	△(32)

√:The same spacers in three isolates; ×: deletions of spacers; △: unique spacers in isolate 21,062; numbers in brackets refer numbers of spacers

It is noteworthy that, compared with isolates 10,010 and 12,038, CRISPR 7 in isolate 21,062 retained the 14 identical spacers on the right side, while 8 spacers on the left were absent (Fig. 6). Spacers in CRISPR arrays are derived from foreign genetic elements in a linear, time-resolved manner [26]. These unique DNA sequences are known to maintain memory against exogenous infection, and the newly obtained DNA (spacer) is located on the 5′ end of the CRISPR arrays [27, 28]. This phenomenon observed in CRISPR 7 in this study indicates that isolate 21,062 has undergone similar infection events to those of the other two isolates in the past, but have diverged in recent evolution, they became divided. In a previous study of the CRISPR-Cas system in *C. difficile*, the CRISPR arrays reached 8.5 arrays/genome [29], however, this number was markedly enriched at 12.5 arrays/genome in our previous study of clade 4 strains (manuscript under reviewed). In the three clade 5 ST11 *C. difficile* isolates in this study, the average number of arrays/genome was 13. CRISPR-Cas genotyping is associated with outbreak tracking, important phenotypes (antibiotic-resistance cassettes), and prophages. Differences among the CRISPR spacers in the closely related isolates in this study reflect the role of CRISPR-Cas systems in controlling the uptake and dissemination of particular genes and operons involved in bacterial adaption and pathogenesis as well as the specific evolution and genotyping of closely related isolates [30]. In this study, large numbers of spacer deletions and acquisitions were identified in the three ST11 group isolates, demonstrating that dynamic changes have occurred in the CRISPR array content. Furthermore, although the three isolates all belongs to ST11 group, unique genetic changes were identified in the spacers in RT 078, suggesting the possibility of distinct interactions with foreign DNA elements within the three isolates. Similarly, in a previous study, stain M120 (RT 078) was shown to possesses the largest number of unique spacers, and also to have hits to a *Clostridium* plasmid [31].

### Antimicrobial susceptibility tests and related drug-resistance genes carried by the three ST11 *C. difficile* isolates

Three of the isolates demonstrated high sensitivity to 11 antibiotics, except isolate 12,038, which was resistant to CIP, and isolate 21,062 which showed intermediate susceptibility to CLI. The hypervirulent RT027 is always associated with fluoroquinolone resistance. In our previous study of clade 4 *C. difficile* isolates, over 90% of the isolates exhibited multi-drug resistance (MDR), and all isolates displayed resistance to CIP (manuscript under reviewed). Surprisingly, all these three isolates were from elderly hospitalized patients undergoing antibiotics treatment [9]. Although the reasons for the high level of antibiotic susceptibility observed in the three isolates in this study are unclear, it can be speculated that the prolonged duration of antibiotic usage might suppress the diversity of the gut microbiota, leading to low rates of horizontal gene transfer by mobile genetic elements, and thereby, reducing the acquisition of antibiotic resistance genes.

**Fig. 8:**
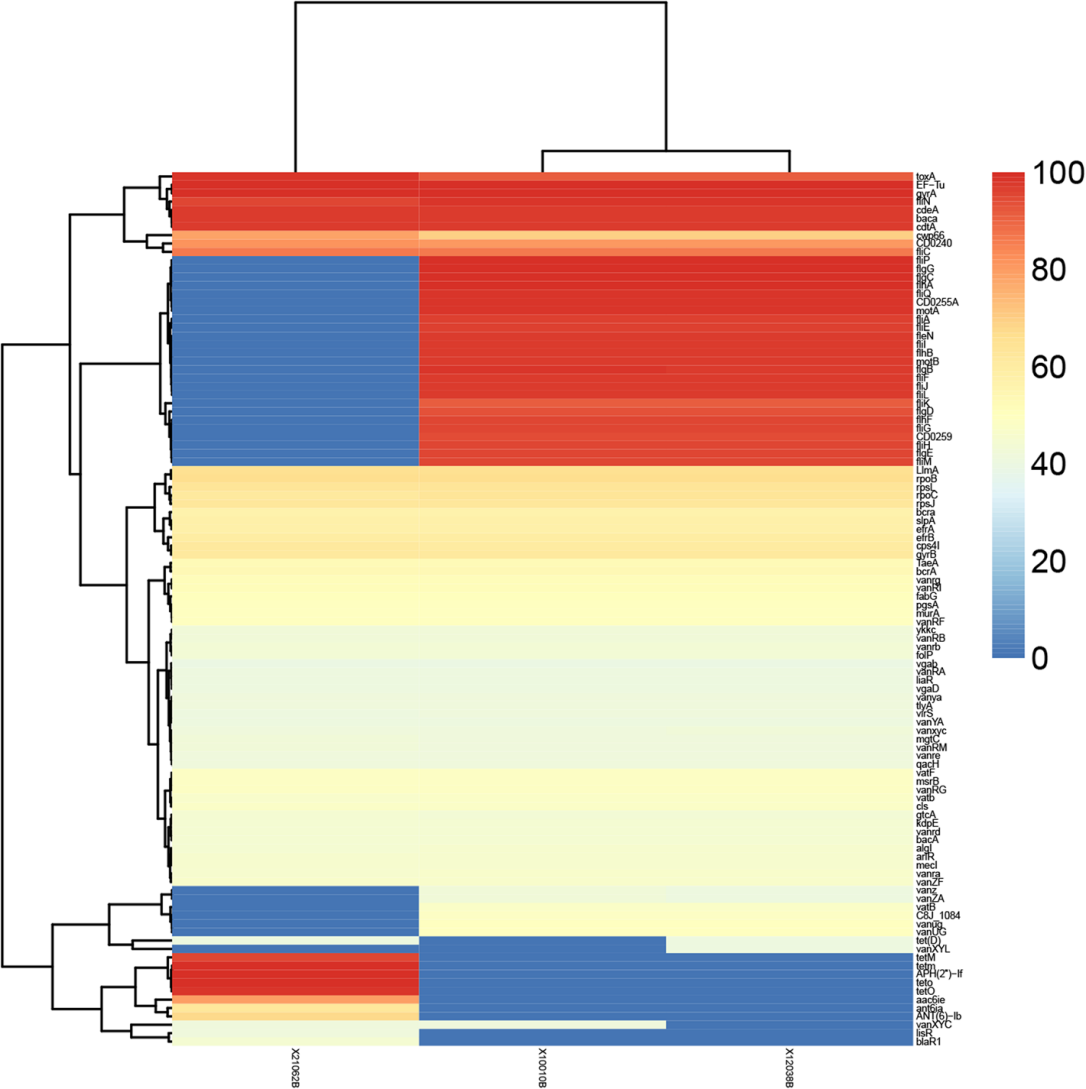
Predicted antibiotic-resistance genes of the three *Clostridium difficile* isolates and virulence-related genes according to the CARD, ARDB and VFDB databases, respectively. The vertical line indicates the predicted genes, and the horizontal lines indicate the three isolates. Increasing numbers of gene copies are represented by the change from blue to red

We explored the antibiotic-resistance and virulence related genes throughout the genomes of the three isolates by comparisons with the CARD, ARDB and VFDB databases (Fig. 8). Isolate 21,062 (RT078/ST11) displayed a unique genes composition with several genes absent or present compared with those of the other two isolates (Fig. 8). A series of genes from *fliP* to *fliM*, which encode proteins related to flagellum structure, biosynthesis and motility, were absent in isolate 21,062 (Fig. 8). In addition, another series of genes predominantly related to vancomycin resistance (*vanZ*, *vanZA*, *vanB*, *vanUG*, and *vanXYL*), were also absent in strain 21,062 (Fig. 8). However, all these three isolates displayed high sensitivity to vancomycin in E-test analysis, which indicates that these genes are not critical elements for VAN resistance, or that they contain non-functional ORFs. A *vanB* operon in Tn*1549* responsible for VAN resistance was originally described in *E. faecalis* [32]. In a recent report, a vanG-like gene cluster, homologous to the cluster found in *E. faecalis*, was described in a number of ST11 *C. difficile* isolates, and although this cluster is expressed, it is still unable to promote resistance to VAN [33]. Furthermore, strain 21,062 also carry specific genes that were absent in the other two isolates, such as *tetD* and *tetO* responsible for TET resistance, and *aac6ie*, and *aac6ia* responsible for resistance to aminoglycoside antibiotics. The gene compositions of isolates 10,010 and 12,038 are almost identical, with the exception of the distribution of three genes (*tetD*, *vanXYL*, and *vanXYC*) (Fig. 8). The effects of the loss of flagellum-related genes on the motility and invasive abilities of isolate 21,062 compared with those of the other two isolates in which the genes are present remains to be investigated.

## Conclusions

This study comprehensively studied the MGEs, antibiotic-resistance genes, and virulent-related genes within ST11 group through the third WGS, which gave insights into the independent microevolution and genome reconstruction of ST11 *C. difficile* isolates. Furthermore, these genetic elements were distinct in RT078 and the other two closely related strains, which might be used as identification and classification markers for RT078. In addition, capillary electrophoresis based PCR-ribotyping is important for confirming RT078, because it carried exactly the same *tcdC* gene with the other two ST11 isolates.

## Methods

### Ethics statement

This study was approved by the Ethics Committee of the National Institute for Communicable Disease Control and Prevention, China CDC. All adult subjects provided informed consent, and no child was involved. The informed consent was given orally because feces collection and further test were standard protocols for patients with diarrhea. And the consent was recorded in daily progress notes by the attending physician at the hospital.

### Isolates and preparation of genomic DNA

Three ST11 *C. difficile* isolates with distinct RTs were isolated from elderly hospitalized patients. Details of these isolates are summarized in Table 1 and our previous study [9]. PCR-ribotyping was performed by capillary electrophoresis using both QIAXcel and ABI 3730 systems [34, 35]. Strains 630 (AM180355) and M120 (FN665653.1) were used as references throughout the investigation. All three isolates were cultured on brain heart infusion (BHI) agar plates (Oxoid, UK) supplemented with 5% sheep blood (BaoTe, China) in an anaerobic chamber (80% nitrogen, 10% hydrogen and 10% carbon dioxide) (Mart, NL) at 37 °C for 48 h. Typical colonies were picked up and re-cultured on BHI for 24 h before preparation of genomic DNA using the Wizard® Genomic DNA Purification Kit (Promega, USA) according to the manufacturer’s instructions.

### Genome sequencing and assembly

The complete genomes of three ST11 *C. difficile* isolates were sequenced on the PacBio RS II platform and Illumina HiSeq 4000 platform at the Beijing Genomics Institute (BGI, Shenzhen, China). Four SMRT cells Zero-Mode Wave guide arrays of sequencing, were used by the PacBio platform to generate the sub-reads set, in which PacBio subreads with length < 1 kb were removed. The program Pbdagcon program (https://github.com/PacificBiosciences/pbdagcon) was used for self-correction. Draft genomic contigs, which are uncontested groups of fragments, were assembled using the Celera Assembler against a high quality corrected circular consensus sequence sub-reads set. To improve the accuracy of the genome sequences, the GATK (https://www.broadinstitute.org/gatk/) and SOAP tool packages (SOAP2, SOAPsnp, SOAPindel) were used to make single-base corrections. To trace the presence of any plasmid, the filtered Illumina reads were mapped using SOAP to the bacterial plasmid database (http://www.ebi.ac.uk/genomes/plasmid.html, last accessed July 8, 2016) using SOAP.

### Genome component prediction

Gene prediction was performed on the three genomes assembled by glimmer3 (http://www.cbcb.umd.edu/software/glimmer/) with a Hidden Markov models. tRNA, rRNA and sRNAs were recognized by using the tRNAscan-SE [36], RNAmmer, and the Rfam database, respectively [37]. The TR annotation was performed using the Tandem Repeats Finder (http://tandem.bu.edu/trf/trf.html). The minisatellite DNA and microsatellite DNAs were selected based on the number and length of the repeat units. Prophage regions were predicted using the PHAge Search Tool (PHAST) web server (http://phast.wishartlab.com/) and CRISPR identification were conducted by using the CRISPRFinder [38].

### Gene annotation and protein classification

The best hit analyzed using BLAST alignment tool for function annotation. The following databases used for general function annotation: KEGG (Kyoto Encyclopedia of Genes and Genomes) [39], COG (Clusters of Orthologous Groups) [40], NR (Non-Redundant Protein Database databases) [41], Swiss-Prot [42], and GO (Gene Ontology) [43], TrEMBL, and EggNOG [44], are used for general function annotation. Four databases were used for pathogenicity and drug-resistance analysis. Virulence factors and resistance genes were identified based on the core dataset in Virulence Factors of Pathogenic Bacteria (VFDB), Antibiotic Resistance Genes Database (ARDB) https://ardb.cbcb.umd.edu/, and Comprehensive Antibiotic Resistance Database (CARD) https://card.mcmaster.ca/. The Pathogen Host Interactions (PHI) and Carbohydrate-Active enzymes (CAZy) databases [45] were also used here.

### Sequence analysis of the PaLoc and CdtLoc regions

The PaLoc and CdtLoc regions were confirmed by comparison to reference CD630 genome. Orthologous gene (> 80% coverage and 90% nucleotide identity) were detected in BLAST (version 2.2.12) searches. The genetic structure as well as insertions and deletions (indel) were also studied.

### Analysis of MGEs

Transposons (Tns) and conjugative transposons (CTns) were identified in BLAST, searches of the *C. difficile* genome sequences or transposons sequences available at NCBI (https://www.ncbi.nlm.nih.gov/). The Tns and CTns were defined as > 80% nucleotide identity and coverage. Prophages were identified using the PHASTER web server (http://phaster.ca/). Intact and incomplete prophages sequences were defined as ≥80% coverage and ≥ 90% highest nucleotide identity with similar regions in bacterial genomes in the databases. To identify plasmids, reads were assembled into contigs using the SOAP denovo. Contigs were screened for plasmids using Microbial Genome BLAST against the NCBI complete plasmid database (ftp://ftp.ncbi.nlm.nih.gov/genomes/refseq/plasmid/). The potential plasmids were defined as ≥70% coverage and ≥ 80% identity. The CRISPRFinder was used for the CRISPR identification. Conserved spacers were used as anchoring points to compare CRISPR arrays across whole genomes. For each array, the repeat sequences were removed, and the list of spacers was oriented with the ancestral spacer on the right-hand side.

### Antimicrobial susceptibility tests

*C. difficile* isolates were tested for susceptibility to moxifloxacin (MXF), vancomycin (VAN), clindamycin (CLI), tetracycline (TET), erythromycin (ERY), rifampin (RFX), levofloxacin (LFX), chloramphenicol (CHL), metronidazole (MTZ), ciprofloxacin (CIP), and meropenem using E-test strips (Biomerieux, France, and Liofilchem, Italy). The interpretation of minimum inhibitory concentration (MIC) for MTZ, MXF, CLI, CIP, LFX, and TET were interpreted according to recommendations of the Clinical and Laboratory Standards Institute (CLSI) M11-A7 and M100-S24 [46, 47], and the European Committee on Antimicrobial Susceptibility Testing (EUCAST) (http://www.eucst.org). The breakpoints for VAN, RFX, ERY, CHL, and meropenem were determined according to a previous study [48]. Multidrug resistance (MDR) was defined as resistance to at least three antimicrobial classes. *C. difficile* ATCC 700057 was included as a control in each experiment.

Antimicrobial resistance genes were predicted through comparison with the Antibiotic Resistance Genes Database (ARDB) https://ardb.cbcb.umd.edu/ [49], and Comprehensive Antibiotic Resistance Database (CARD) https://card.mcmaster.ca/ databases [50]. Heatmap analysis was performed using the pheatmap package and stats packages in R software (version 2.15.3).

### Nucleotide sequence accession numbers

The complete whole-genome sequences of 3 ST11 *C. difficile* isolates have been submitted to DDBJ/EMBL/GenBank under the BioProject number PRJNA497978.

## Supplementary information

**Additional file 1. **Table summarizing transposals identified in the three *C. difficile* isolates.

## Data Availability

The data generated and/or analyzed during the current study are available from the corresponding author on reasonable request.

## Abbreviations

CDs: Coding sequences
CRISPR: Clustered regularly interspersed short palindromic repeat
HLGR: High level gentamicin resistance
MGEs: Mobile genetic elements
MLST: Multi locus sequencing typing
ncRNAs: non-coding RNAs
ORF: Open reading frame
RTs: Ribotypes
SNPs: Single nucleotide polymorphisms
TRs: Tandem repeats
WGS: Whole genome sequencing
